# [Retracted] Long non‑coding RNA SLC25A25‑AS1 exhibits oncogenic roles in non‑small cell lung cancer by regulating the microRNA‑195‑5p/ITGA2 axis

**DOI:** 10.3892/ol.2024.14608

**Published:** 2024-08-05

**Authors:** Jinqin Chen, Chengpeng Gao, Wei Zhu

Oncol Lett 22: 529, 2021; DOI: 10.3892/ol.2021.12790

Following the publication of this paper, it was drawn to the Editor's attention by a concerned reader that, in addition to concerns about the presentation of the western blots and tumor images in this paper, certain of the Transwell migration and invasion assay data shown in Fig. 5C on p. 9 were strikingly similar to data that had already been published in different form in another article written by different authors at different research institutes.

Owing to the fact that the contentious data in the above article had already been published prior to its submission to *Oncology Letters*, the Editor has decided that this paper should be retracted from the Journal. The authors were asked for an explanation to account for these concerns, but the Editorial Office did not receive a reply. The Editor apologizes to the readership for any inconvenience caused.

